# Slr0320 Is Crucial for Optimal Function of Photosystem II during High Light Acclimation in *Synechocystis* sp. PCC 6803

**DOI:** 10.3390/life11040279

**Published:** 2021-03-26

**Authors:** Hao Zhang, Haitao Ge, Ye Zhang, Yingchun Wang, Pengpeng Zhang

**Affiliations:** 1 Biotechnology Research Institute, Chinese Academy of Agricultural Sciences, Beijing 100081, China; zhanghaocaas@163.com (H.Z.); zhangyecaas@163.com (Y.Z.); 2 Institute of Genetics and Developmental Biology, Chinese Academy of Sciences, Beijing 100101, China; htge@genetics.ac.cn (H.G.); ycwang@genetics.ac.cn (Y.W.)

**Keywords:** high light acclimation, photosystem II, electron transfer between Q_A_ and Q_B_, low molecular weight subunits, quantitative proteomics, *Synechocystis* sp. PCC 6803

## Abstract

Upon exposure of photosynthetic organisms to high light (HL), several HL acclimation responses are triggered. Herein, we identified a novel gene, *slr0320*, critical for HL acclimation in *Synechocystis* sp. PCC 6803. The growth rate of the Δ*slr0320* mutant was similar to wild type (WT) under normal light (NL) but severely declined under HL. Net photosynthesis of the mutant was lower under HL, but maximum photosystem II (PSII) activity was higher under NL and HL. Immunodetection revealed the accumulation and assembly of PSII were similar between WT and the mutant. Chlorophyll fluorescence traces showed the stable fluorescence of the mutant under light was much higher. Kinetics of single flash-induced chlorophyll fluorescence increase and decay revealed the slower electron transfer from Q_A_ to Q_B_ in the mutant. These data indicate that, in the Δ*slr0320* mutant, the number of functional PSIIs was comparable to WT even under HL but the electron transfer between Q_A_ and Q_B_ was inefficient. Quantitative proteomics and real-time PCR revealed that expression profiles of *psbL*, *psbH* and *psbI* were significantly altered in the Δ*slr0320* mutant. Thus, Slr0320 protein plays critical roles in optimizing PSII activity during HL acclimation and is essential for PSII electron transfer from Q_A_ to Q_B_.

## 1. Introduction

Light is an essential environmental factor for all photosynthetic organisms, including higher plants, algae and cyanobacteria. Photosynthetic organisms perform photosynthesis by converting absorbed sunlight energy into chemical energy. Photosystem II (PSII), as the first enzyme of photochemistry, plays essential roles in photosynthesis. The PSII is a multi-subunit protein complex embedded in the thylakoid membrane, catalyzing water oxidation, plastoquinone reduction and oxygen release [1,2,3]. The reaction center (RC) of PSII consists of heterodimeric D1 and D2 proteins that are flanked by chlorophyll-binding proteins CP43 and CP47. Upon absorbing light, light-driven charge separation occurs between the P680 and pheophytin in the RC [4,5]. The charge-separated electron could in turn transfer to two quinone acceptors, Q_A_ and Q_B_. The oxygen evolution complex (OEC), harboring a manganese cluster as well as PsbO, PsbU and PsbV in cyanobacteria, is located at the lumen side of PSII and catalyzes the water-splitting, releasing oxygen. In addition to the subunits mentioned above, PSII contains 13 subunits with molecular weights less than 10 kDa, known as low molecular weight (LMW) subunits (PsbE, PsbF, PsbH, PsbI, PsbJ, PsbK, PsbL, PsbM, PsbN, PsbT, PsbX, PsbY, PsbZ) in *Synechocystis* sp. PCC 6803 (hereafter *Synechocystis*). These LMW subunits are thought to play important roles in PSII biogenesis, structure, assembly and electron transfer [6,7,8].

Light is necessary for photosynthesis, but it can cause damages and become dangerous as well. Given that the light environment for photosynthetic organisms is constantly changing, the situation becomes more complicated. The D1 protein of PSII is the main target of the light-induced damages and these unavoidable damages are positively correlated to light intensities [9,10]. When the light intensities increase and the imbalance between the PSII photodamage and PSII repair occurs, the photoinhibition become apparent [11]. *Synechocystis* has developed several high light (HL) acclimation strategies to alleviate photoinhibition, such as the upregulation of D1 encoding genes [12,13,14], highly efficient proteolytic degradation of damaged D1 [15,16,17], elevated levels of specific carotenoids and translation elongation factors [18,19], and specific flavodirron proteins [20]. Owning to deeper investigations of *Synechocystis* gene functions, more and more hypothetical and known genes have been identified as being involved in the HL acclimation and protection of PSII, such as *slr0151* [21] and five small CAB-like proteins (ScpA-E), of which ScpB-E are also known as high light-induced proteins (HliA-D) [22,23].

This process is not completely clear, although many efforts and some progresses have been made in discovery of novel genes involved in HL acclimation. To investigate new genes involved in HL acclimation, a random transposon-insertional mutant library of *Synechocystis* was screened. A novel gene, *slr0320*, was identified as being involved in HL acclimation and optimal function of PSII. The possible mechanism of the *slr0320* involved in HL acclimation was discussed.

## 2. Materials and Methods

### 2.1. Culture Conditions

The culture conditions were set according to Zhang et al. [24]. The *Synechocystis* sp. PCC 6803 glucose-tolerant strain (used as wild type, WT) [25], the Δ*slr0320* mutant and complementary strain P*petJ*::*slr0320* were grown either in liquid or on solid BG-11 medium buffered with 20 mM HEPES-NaOH (pH 7.5) at 30 °C. The solid BG-11 medium was supplemented with 1.5% agar. The liquid cultures were aerated by gentle shanking at 120 rpm. The cells were illuminated under continuous white LED light (4100 K) with photon flux density of either 50 μmol photons m^−2^ s^−1^ (normal light, NL) or 220 μmol photons m^−2^ s^−1^ (high light, HL). All mutant strains were maintained in the presence of appropriate antibiotics.

### 2.2. Mutant Construction and Screening

The *Synechocystis* mutant library was constructed according to Ozaki et al. [26]. The liquid wild type cells were transformed with the transposon mutagenesis library, which contains a chloramphenicol-resistant (Cm^R^) cassette. Following transformation, Cm^R^ mutants showing slower growth rates under HL but normal growth rates under NL on solid plates were selected. Genomic DNA extracted from selected mutants were digested by *Hha*I and, after self-ligation, used as templates for PCR with primers (P4) complementary to two ends of the chloramphenicol-resistant cassette (Table 1). The exact genome positions of insertions of transposons with Cm^R^ were determined by sequencing PCR products.

Due to disruptions by a frameshift, *spkA* (*sll1574*–*sll1575*) is considered as a neutral site in *Synechocystis* [27]. The complementary strain P*petJ*::*slr0320* was constructed according to Eisenhut et al. [28]. The wild type copy of *slr0320* gene driven by a *petJ* promoter was integrated into the *spkA* site of genome in the Δ*slr0320* mutant.

### 2.3. RNA Isolation and Quantification

Total RNAs were isolated by Trizol method based on Zhang et al. [29]. After removal of genomic DNA by TURBO DNA-free Kit (Invitrogen, Carlsbad, CA, USA), the first strand cDNA was synthesized from 2.5 μg total RNA using a Revert aid First Strand cDNA Synthesis Kit (Thermo Scientific, Carlsbad, CA, USA). Quantitative Real-Time PCR (qRT-PCR) was performed on an ABI (Applied Biosystems Inc., Foster City, CA, USA) QuantStudio 6 Flex system. The primers for analysis were *psbH* (*ssl2598*), *psbI* (*sml0001*), *psbM* (*sml0003*), *psbK* (*sml0005*), *psbX* (*sml0002*), *psbZ* (*sll1281*), *psbJ* (*smr0008*), *psbN* (*smr0009*) and *psbT* (*smr0001*) transcripts, including the reference gene *rnpB* (*slr0249*), as listed in Table 1. Due to some of target genes being too short, the primers were designed to generate a similar length (~100 bp) of amplicons. The relative changes in gene expressions were calculated by the 2^−ΔΔCt^ method [30].

### 2.4. Protein Isolation, Electrophoresis, and Immunodetection

The total cell extract as well as membrane and soluble fractions were isolated according to Zhang et al. [31]. Cells from liquid cultures were harvested at 5000 rpm, 4 °C for 5 min and washed with washing buffer (50 mM HEPES-NaOH pH 7.5, 30 mM CaCl_2_). The harvested cells were then resuspended in resuspension buffer (50 mM HEPES-NaOH pH 7.5, 30 mM CaCl_2_, 800 mM sorbitol and 1 mM 6-aminohexanoic acid) and broken in the presence of glass beads (150–212 μm, Sigma, St. Louis, MO, USA) by manually vortexing eight times at maximal speed for 1 min at 4 °C with 1 min cooling on ice between cycles. After removal of glass beads and cell debris at 2000× *g* for 5 min at 4 °C, the total cell extract was obtained. The total cell extract, which contains membrane and soluble fractions, could be further separated by centrifugation at 30,000× *g* for 30 min at 4 °C. After centrifugation, the membrane was pelleted down. Additionally, the soluble fraction remained in supernatant. The pelleted membrane was resuspended in storage buffer (50 m HEPES-NaOH pH 7.5, 30 mM CaCl_2_, 600 mM sucrose and 1 M betaine), and directly used for further analysis or frozen by liquid nitrogen in aliquots.

Protein samples were separated and immunodetected according to Zhang et al. [24]. Membrane samples for BN-PAGE (blue native-polyacrylamide gel electrophoresis) were performed as follows. The membrane samples were washed with washing buffer (50 mM Bis-Tris pH 7.0, 330 mM sorbitol) supplemented with 1 mM AEBSF (Thermo Scientific, Carlsbad, CA, USA) which is a protease inhibitor and pelleted by centrifugation at 30,000× *g* for 10 min at 4 °C. The pellets were then suspended in the buffer containing 25 mM Bis-Tris pH 7.0, 1 mM AEBSF, 20% glycerol (*w*/*v*) and 10 mM MgCl_2_ with chlorophyll concentration at 1 μg/μL. The protein samples were further solubilized by gently adding an equal volume of 3% *n*-dodecyl-β-D-maltoside to the same buffer, followed by 20 min incubation on ice. Insoluble material was removed by centrifugation at 30,000× *g* for 15 min at 4 °C. The collected supernatant was mixed with 1/10 volume sample buffer containing 5% Serva blue G (*w*/*v*), 100 mM Bis-Tris, pH 7.0, 30% sucrose (*w*/*v*), and 0.5 M 6-aminohexanoic acid and loaded onto 5 to 12.5% acrylamide gradient gel. Electrophoresis for BN-PAGE was performed at 4 °C by increasing voltage gradually from 50 up to 200 V during the 5.5 h run. Protein samples for SDS-PAGE (sodium dodecyl sulfate- polyacrylamide gel electrophoresis) were solubilized in Laemmli SDS sample buffer [32] containing 5% β-mercaptoethanol and 6 M urea at room temperature for 1h and then separated by 12% SDS-PAGE with 6 M urea.

After electrophoresis, the proteins were electrotransferred to a polyvinylidene fluoride (PVDF) membrane (Immobilon-P, Millipore, Burlington, MA, USA) by a semidry apparatus and immunodetected with specific antibodies. NdhK and NdhH antibodies were kind gifts from Weimin Ma from Shanghai Normal University and Hualing Mi from Institute of Plant Physiology and Ecology, Chinese Academy of Sciences, respectively. FNR (ferredoxin: NADP^+^ oxidoreductase) antibody was from Ghada Ajlani from Centre National de la Recherche. The D1, D2, CP43, CP47, PsbO, PsaA, RbcL, PetC and AtpB antibodies were purchased from Agrisera (Vennes, Sweden).

### 2.5. Oxygen Evolution Measurements

The steady-state oxygen evolution rate was measured by a Clark-type oxygen electrode (Hansatech) at saturating white light intensity (2500 μmol photons m^−2^ s^−1^) according to Zhang et al. [33]. The cells of WT, Δ*slr0320* and P*petJ*::*slr0320* strains grown in liquid BG-11 medium under NL and HL were harvested and resuspended in fresh BG-11 medium to chlorophyll concentration of 5 µg/mL. Net photosynthesis was measured in the presence of 10 mM NaHCO_3_. PSII activities were measured in the presence of 0.5 mM DCBQ (2,6-dichloro-*p*-benzoquinone) and 1 mM K_3_Fe(CN)_6_.

### 2.6. Chlorophyll Fluorescence Measurement

The chlorophyll fluorescence traces were measured by a Joliot-type spectrophotometer, Jts-10 (Biologics). Cells with chlorophyll concentration of 5 µg/mL were dark-adapted for 15 min, illuminated with either 45 μmol photons m^−2^ s^−1^ or 320 μmol photons m^−2^ s^−1^ orange actinic light (630 nm), followed by recovery in darkness. Saturating pulses (7900 μmol photons m^−2^ s^−1^, 200 ms duration) were applied to measure maximum fluorescence Fm and Fm’.

Flash-induced fluorescence increase and the subsequent decay of chlorophyll fluorescence yield were performed on a fluorometer FL 3500 (PSI Instruments) according to Zhang et al. [20]. The cells of WT, Δ*slr0320* and P*petJ*::*slr0320* strains, grown under NL and HL, were harvested and resuspended to chlorophyll concentration of 5 µg/mL. After 10 min dark adaption, cells were excited with a single, short saturation flash-light. According to Cser et al. [34], a fitting function with three components was applied:

F(t) − F_0_ = A1 × exp(−t/T1) + A2 × exp(−t/T2) + A3/(1 + t/T3) (1)

Equation (1) was used for deconvolution of the measured curves, where F(t) is the variable fluorescence yield at time t, F_0_ is the basic fluorescence level before the flash, A1 to A3 are the amplitudes, and T1 to T3 are the time constants. The Joliot model [35] was used to correct the nonlinear correlation between fluorescence yield and the redox state of Q_A_ with a value of 0.5 for the energy transfer parameter between the PSII units [20,36].

### 2.7. Quantitative Proteomics Analysis

The protein preparation was performed according to Ge et al. [37]. The harvested cells were broken by a bead beater. The total cell extract was precipitation with ice-cold 10% trichloroacetic acid in acetone at −20 °C, washed with acetone, and resolubilized with 4% sodium dodecyl sulfate (SDS) in 0.1 M Tris-HCl, pH 7.6. The BCA protein assay kit (Thermo Scientific, Rockford, IL, USA) was used to determine the protein concentration. The filter-aided sample preparation (FASP) method [38] was used to digest total proteins to tryptic peptides. Briefly, DTT (dithiothreitol) (100 mm) and iodoacetamide (55 mm) were used for reduction and alkylation of the sample proteins and then the reduced proteins were transferred into the Microcon YM-30 centrifugal filter unites to exchange the denaturing buffer with triethylammonium bicarbonate (TEAB, 0.1 M) buffer. Proteins were then digested with trypsin (Promega, Madison, WI, USA) at 37 °C overnight, and the tryptic peptides were labeled with TMT reagents (Thermo Scientific, Rockford, IL, USA) by incubation at room temperature in dark for 2 h. The labeling process was stopped with 5% hydroxylamine and the equal amounts of each labeled samples were mixed together for next prefractionation with reversed phase (RP) high performance liquid chromatography (HPLC). After RP and HPLC, 4 fractions were collected and dried by a SpeedVac concentrator and stored at −20 °C for LC–MS/MS analysis. The LC–MS/MS analysis was performed using an Orbitrap Lumos mass spectrometer (Thermo Scientific, Rockford, IL Waltham, MA, USA) coupled online to an Easy-nLC 1000 in the data-dependent mode. The database search for the raw MS files was performed using the software MaxQuant (version 1.6.1, Max Planck Institute of Biochemistry, Martinsried, Germany) [39]. The *Synechocystis* proteome sequence database which contains 3672 entries was downloaded from CyanoBase. Bioinformatics and statistical analyses were performed using the software Perseus (version 1.5.4.1, Max Planck Institute of Biochemistry, Martinsried, Germany) [40], Origin2018 (version b9.5.1.195, OriginLab Corp., Northampton, MA, USA).

## 3. Results

### 3.1. Identification of slr0320 under High Light

To identify genes involved in high light acclimation, a *Synechocystis* transposon-insertion mutant library was screened by comparison of the growth under high light (HL, 220 μmol photons m^−2^ s^−1^) and normal light (NL, 50 μmol photons m^−2^ s^−1^). By screening approximately 1000 mutants, 90 mutants were identified as photosensitive. Among identified mutants, a mutant showing significantly retarded growth under HL but normal growth under NL was selected for further analysis.

**Figure 1:**
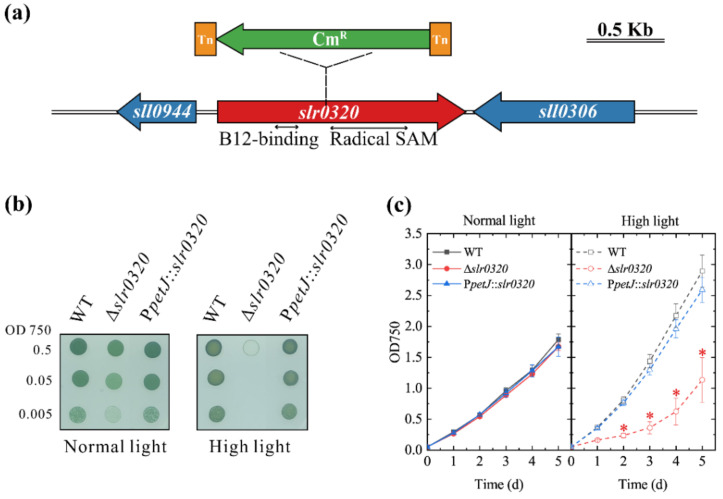
Gene structure of *slr0320* and growth of wild type (WT), Δ*slr0320* and P*petJ*::*slr0320* strains. (**a**) Organization of *slr0320* in *Synechocystis*. The insertion position of a transposon including a chloramphenicol-resistant cassette (Cm^R^, green) flanked by two insertion sequences (Tn, orange) is indicated; two conserved domains of *slr0320* were indicated under the *slr0320* coding region (red); (**b**) growth of WT, Δ*slr0320* and P*petJ*::*slr0320* strains on agar plates under normal light (NL) and high light (HL). The cells grown under NL were harvested and resuspended in fresh BG-11 medium. Then, they were diluted to 0.5, 0.05, 0.005 at OD750 and 10 μL liquid cells were placed on the agar plate. The pictures were taken after 6 days; (**c**) growth curves of WT, Δ*slr0320* and P*petJ*::*slr0320* strains in liquid medium under NL and HL. The initial OD750 was 0.05. Student’s *t* test was applied. Red asterisks mark significant differences for the Δ*slr0320* mutant vs. WT and P*petJ*::*slr0320* strains, with a significance level of *p* < 0.05. The results are plotted as the means from three independent experiments ± SD.

PCR sequencing data showed that the transposon insertion occurred at position 709 of *slr0320* (the A of the start codon ATG was set as position 1). The *slr0320* gene is 1596 bp in length and consists of two conserved domains: a B12-binding domain from position 373 to 528 and a radical *S*-adenosyl methionine (SAM) domain from position 724 to 1209 (Figure 1a).

To confirm the phenotype of the mutant was caused by the defect in *slr0320* gene, a complementary strain, P*petJ*::*slr0320,* was constructed. The WT, Δ*slr0320* and P*petJ*::*slr0320* strains were grown on BG-11 agar plate under HL and NL. Under NL conditions, there were no apparent differences among WT, the Δ*slr0320* mutant and the complementary strain P*petJ*::*slr0320*. However, under HL condition, the Δ*slr0320* mutant was grown much slower than WT and the P*petJ*::*slr0320* strain (Figure 1b). The growth phenotypes of the cells grown in liquid cultures were similar to that grown on agar plate. Under NL, growth rates of WT, Δ*slr0320* and the P*petJ*::*slr0320* strain were similar. Under HL, growth rates of WT and the P*petJ*::*slr0320* strain were similar and increased, but the growth rate of the Δ*slr0320* mutant was significantly inhibited (Student’s *t* test, *p* < 0.05) (Figure 1c).

To clarify the reason for the retarded growth rate of the Δ*slr0320* mutant, the steady-state oxygen evolution rates of net photosynthesis and PSII activity were monitored. When cells were grown under NL, there was no significant difference of maximal net photosynthesis among WT, Δ*slr0320* and P*petJ*::*slr0320* strains (Student’s *t* test, *p* < 0.05). However, about 23% decline in the Δ*slr0320* mutant was observed when cells were grown under HL. It should be noted that the net photosynthesis rate of the Δ*slr0320* mutant grown under HL was similar to that under NL, while the rates of WT and P*petJ*::*slr0320* strains were significantly increased in HL grown cells (Student’s *t* test, *p* < 0.05), from 311 to 435 μmol O_2_ mg^−1^ chlorophyll h^−1^ and 330 to 444 μmol O_2_ mg^−1^ chlorophyll h^−1^, respectively. However, when supplemented with artificial quinone acceptor 2,6-dichloro-*p*-benzoquinone (DCBQ), the PSII activity of the Δ*slr0320* mutant was even slightly higher than WT and the P*petJ*::*slr0320* strain under both HL and NL (Table 2).

### 3.2. Accumulation of Major Thylakoid Proteins and PSII Complexes in WT, Δslr0320 and PpetJ::slr0320 Strains

**Figure 2:**
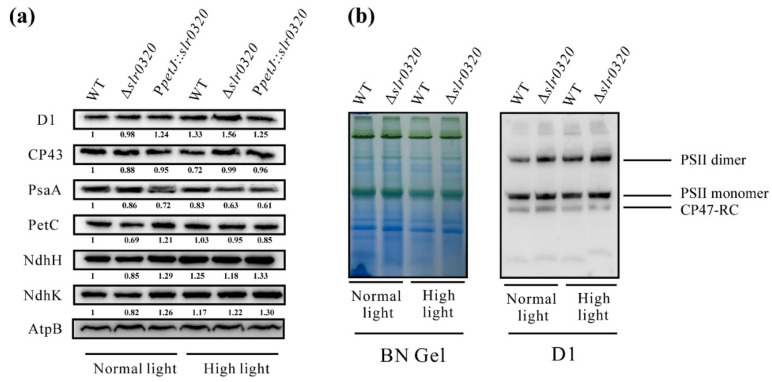
Major thylakoid proteins under NL and HL. (**a**) Thylakoid membrane proteins (5 μg) were separated by SDS-PAGE and immuno-detected by specific antibodies against D1, CP43, PsaA, PetC, NdhH, NdhK and AtpB. The gray scale levels of bands from immunoblots were semiquantified. The protein levels semiquantified by band density below were normalized with AtpB and each WT under NL was set as 1; (**b**) photosystem II (PSII) complexes from thylakoid membrane fraction were separated by BN-PAGE and immunoblotted with D1 protein specific antibody.

The lower oxygen evolution of net photosynthesis of the Δ*slr0320* mutant suggested some modifications of photosynthetic machinery in the mutant. Therefore, the photosynthetic protein contents on the thylakoid membrane in WT, Δ*slr0320* and P*petJ*::*slr0320* strains were analyzed. Western blots of representatives of the major thylakoid membrane protein complexes from the thylakoids isolated from WT, Δ*slr0320* and P*petJ*::*slr0320* strains grown under NL and HL revealed that the differences in amounts of the thylakoid proteins tested were tiny (fold change > 1.4 and Student’s *t* test *p* < 0.05), if any—probably a slight increase in PSII proteins and decrease in PSI in the Δ*slr0320* mutant under HL, a slight decline of cytochrome *b*_6_*f* in the Δ*slr0320* mutant under both NL and HL and almost unaltered NDH-1M complex (Figure 2a).

In addition to the slightly increase in PSII proteins, it is worth noting that the amount of native PSII complexes in the Δ*slr0320* mutant showed similar behaviors under both NL and HL. The BN-PAGE followed by immunodetection against D1 showed that the amounts of PSII dimers, monomers, and CP43-less monomer (CP47-RC), which is the PSII assembly intermediate containing the CP47 subunit, in the Δ*slr0320* mutant were no less than in WT both under NL and HL (Figure 2b).

### 3.3. Chlorophyll Fluorescence in WT, Δslr0320 and PpetJ::slr0320 Strains

**Figure 3:**
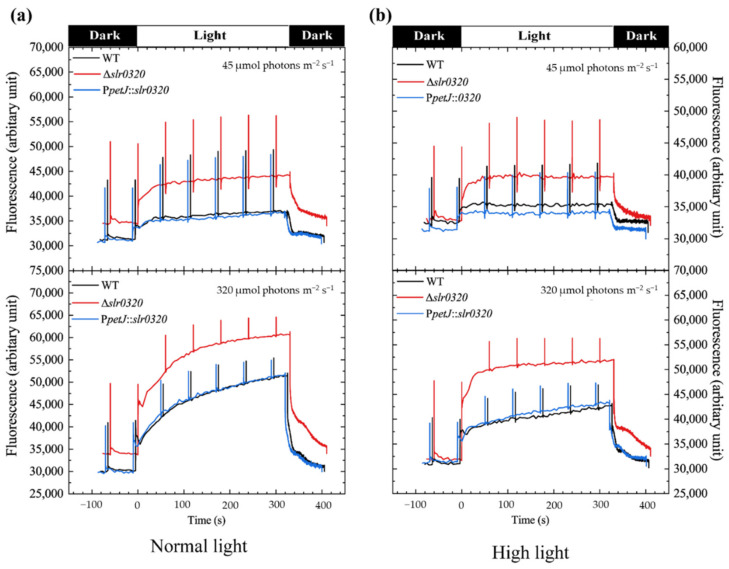

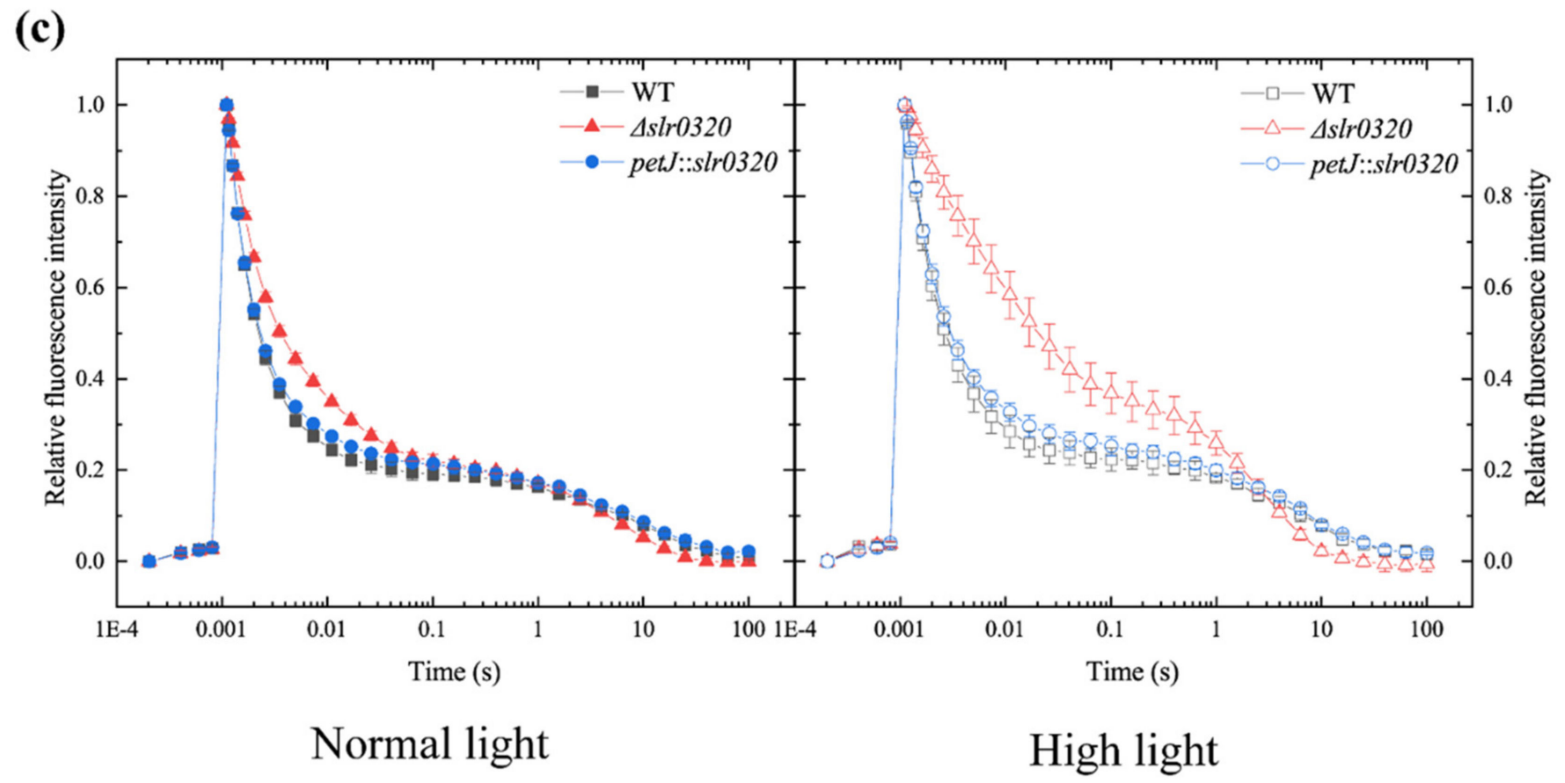
Chlorophyll fluorescence of WT, Δ*slr0320* and P*petJ*::*slr0320* strains. (**a**) Chlorophyll fluorescence traces of the cells grown under NL during dark and light adaptations; (**b**) Chlorophyll fluorescence traces of the cells grown under HL during dark and light adaptations; (**c**) single flash-induced chlorophyll fluorescence curves. The results are plotted as the means from three independent experiments ± SD.

To monitor photosynthetic electron transfer, chlorophyll fluorescence of WT, Δ*slr0320* and P*petJ*::*slr0320* cells grown under NL and HL was monitored. When cells were grown under NL, the dark-adapted initial fluorescence, Fo, was slightly higher in the Δ*slr0320* mutant. Additionally, the light-adapted fluorescence, Fs, remained higher than WT and the complementary strain P*petJ*::*slr0320,* either illuminated with 45 or 320 µmol photons m^−2^ s^−1^ actinic light. When cells were grown under HL, the Fo of the Δ*slr0320* mutant was comparable to WT and the P*petJ*::*slr0320* strain, while the Fs became higher than WT and the P*petJ*::*slr0320* strain under 45 µmol photons m^−2^ s^−1^ actinic light and the difference became a little more apparent when the actinic light was increased to 320 µmol photons m^−2^ s^−1^. Under both NL and HL, the variable fluorescence Fv in the Δ*slr0320* mutant was higher than that of WT and the P*petJ*::*slr0320* strain (Figure 3a,b).

To monitor Q_A_^−^ reoxidation, a single flash induced an increase in chlorophyll fluorescence and the following decay was measured. The fluorescence decay consists of three components, reflecting three pathways of Q_A_^−^ reoxidation. The fast phase and middle phase reveal reoxidation by bounded PQ (plastoquinone) and free PQ, respectively. The slow phase is caused by charge recombination [36]. The chlorophyll fluorescence decay showed that electron transport processes at the acceptor side of PSII was modified in the Δ*slr0320* mutant (Figure 3c). Kinetics analysis demonstrated that apparent slower fluorescence decay in the Δ*slr0320* mutant than WT and the complementation strain P*petJ*::*slr0320* was mainly due to the decrease in the fast phase amplitude and the higher time constants of the fast phase and middle phase (Table 3). In addition, the slow phase in the Δ*slr0320* mutant had smaller time constant and higher amplitude than WT and the P*petJ*::*slr0320* strains. The phenomenon became more evident when the cells were grown under HL (Figure 3c and Table 3).

### 3.4. Quantitative Proteomics of WT and the Δslr0320 Mutant

**Figure 4:**
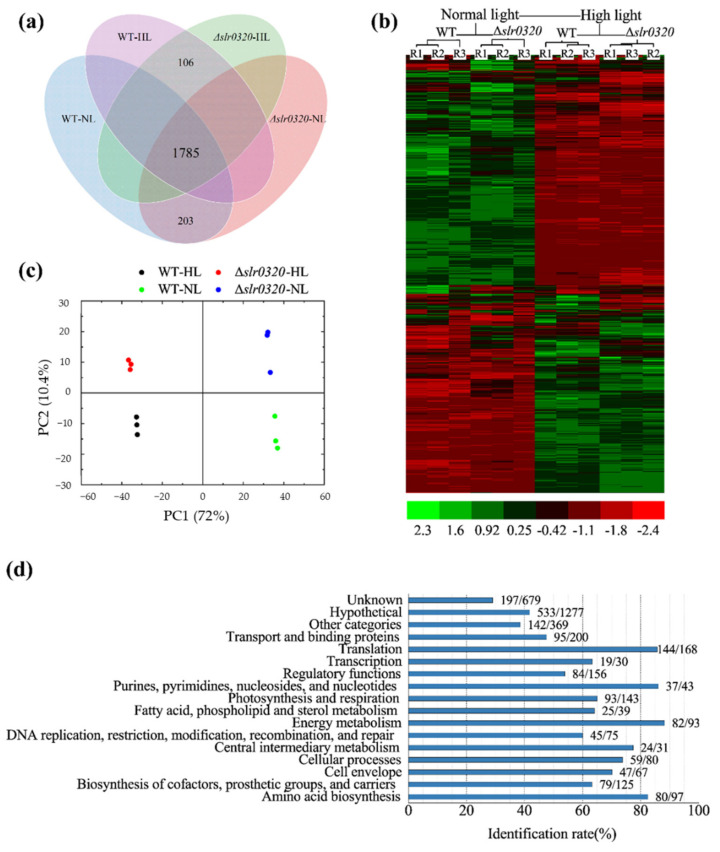
Overview of quantitative identification of proteomes of WT and the Δ*slr0320* mutant. (**a**) Venn diagram showing overlapped proteins. Cells grown under NL are indicated as WT-NL and Δ*slr0320*-NL; cells grown under HL are indicated as WT-HL and Δ*slr0320*-HL; (**b**) hierarchy clustering analysis and heatmap of z-scored report ion intensities. The extents of differential expression are color-coded and shown by the scale bar on the bottom of heatmap. Each sample contained three biological replicates indicated as R1, R2 and R3; (**c**) principal component analysis of all samples. Black dots are WT grown under HL; green dots are WT grown under NL; red dots are Δ*slr0320* cells grown under HL and blues dots are Δ*slr0320* cells grown under NL; (**d**) bar graph showing the coverage of protein identification for all functional categories annotated by the CyanoBase. The numbers to the right of bars are indicated as protein numbers identified in this report/protein numbers in CyanoBase.

To elucidate how Slr0320 operates in HL acclimation, quantitative proteomics was performed on WT and the Δ*slr0320* mutant grown under NL and HL. For cells grown under NL, 1988 proteins were shared by WT and the Δ*slr0320* mutant. When cells were grown under HL, the number of identified proteins was 1891. Combining these four samples, the number of overlapped proteins was 1785 (Figure 4a and Table S1). Hierarchical clustering analysis of the 1785 proteins using z-scored report ion intensities showed that three biological replicates of WT and the Δ*slr0320* mutant, each grown under NL and HL, were clustered (Figure 4b). Principal component analysis also revealed that each sample containing three biological replicates was successfully separated from others (Figure 4c). These two analyses confirmed the high reproducibility of the 1785 overlapped proteins. Grouping these proteins according to the functional categories annotated by the CyanoBase [41] showed that coverages of 1785 proteins for all functional categories were almost more than 50% except for the “other categories”, “unknow” and “hypothetical” (Figure 4d). Taken together, the high reproducibility and coverage ensured the proteome data were suitable for further analysis.

**Figure 5:**
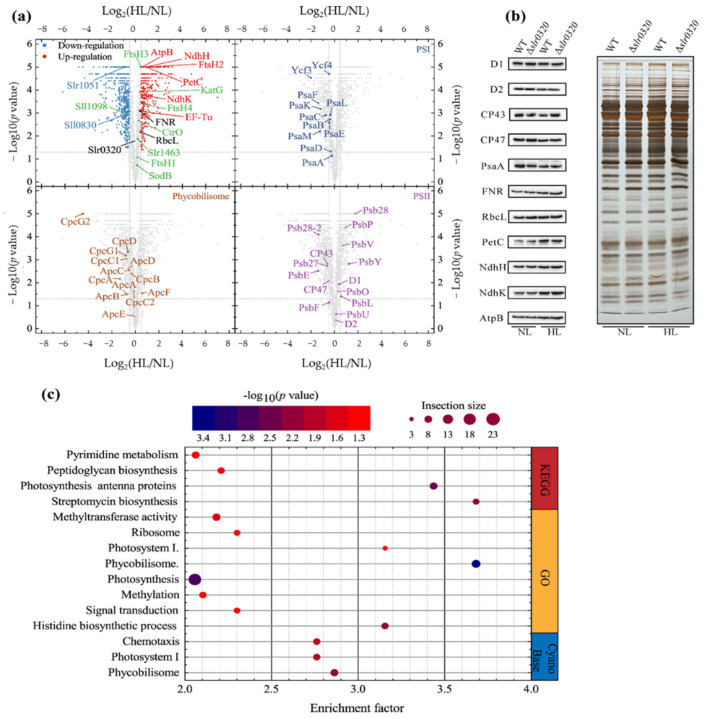
Expressions of proteins in *Synechocystis* WT cells grown under NL and HL. (**a**) Volcano plots showing differentially expressed proteins in *Synechocystis* WT cells grown under NL and HL; (**b**) confirmation of proteome by Western blots of selected proteins. The immunodetections against D1, D2, CP43, CP47, PsbO, PsaA, FNR, RbcL, PetC, NdhH, NdhK and AtpB are on the left panel; the visualization of the whole cell lysate by silver-staining is shown by the right panel; (**c**) bubble plot showing enrichment of significantly downregulated proteins by Fisher’s exact test. The bubble size indicates the number in each term; bubble color indicates the *p* value as shown in the scale bar above the plot.

Before uncovering roles of *slr0320* in HL acclimation, proteins involved in HL acclimation of *Synechocystis* were roughly investigated. The cutoffs of a fold change of 1.4 and *p* < 0.05 (Student’s *t* test) were applied to filter expressed proteins with significant differences and high confidence. Compared to *Synechocystis* grown under NL, 551 proteins were downregulated and 389 proteins were upregulated in WT grown under HL (Figure 5a and Table S2). A Fisher’s exact test was performed to enrich downregulated proteins in gene ontology (GO), KEGG (Kyoto Encyclopedia of Genes and Genomes) pathways and the functional groups annotated by CyanoBase (Figure 5c and Table S5). The result indicated that most of photosynthesis-related proteins were enriched. The majorities of PSI and phycobilisome were downregulated. Among all identified PSI subunits, PsaC (Ssl0563), PsaF (Sll0819), PsaK (Sll0629), PsaM (Smr0005) and Ycf3 (Slr0823) were significantly downregulated. Except for CpcB (Sll1577), CpcC2 (Sll1579), ApcE (Slr0335) and ApcF (Slr1459), the identified phycobilisome subunits were significantly downregulated. Unlike PSI and phycobilisome, subunits of PSII showed different behaviors. CP43 (Sll0851), PsbE (Ssr3451), Psb27 (Slr1645) and Psb28-2 (Slr1739) were significantly downregulated, whereas PsbP (Sll1418), PsbL (Smr0007), PsbY (Sml0007), PsbV (Sll0258) and Psb28 (Sll1398) were significantly upregulated (Figure 5a). Most of these changes were similar to previous transcriptomic data of *Synechocystis* [12,13,14]. Likewisely, most translational behaviors of identified genes in our assay were positively correlated with those in HL-treated *Synechococcus* sp. PCC 7002 [42]. Furthermore, our immune-detection also confirmed selective proteins (Figure 5b). Previous reports showed that some factors involved in PSII photorepair, such as FtsH2 (Slr0228) [16], EF-Tu (Sll1099) [19], Psb28 and Psb27 [43], would increase with elevated light intensity to cope with photoinhibition. Our proteomics also identified their significant upregulations in WT grown under HL (Figure 5a). Thus, our proteomic data could be considered as highly confident. Additionally and unexpectedly, the Slr0320 was almost unaltered (Figure 5a).

**Figure 6:**
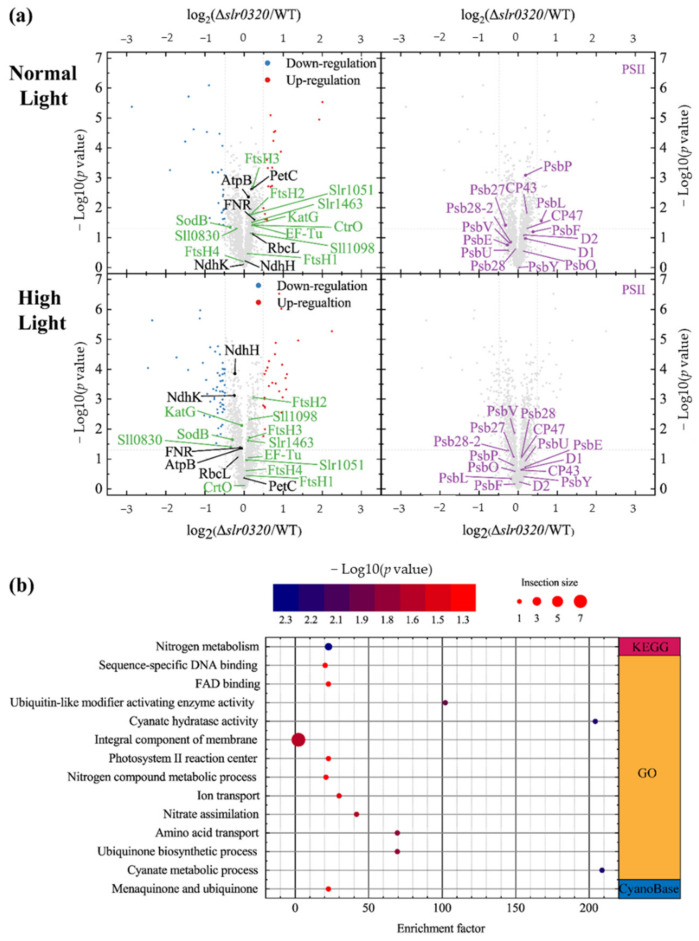
Expressions of proteins in WT and the Δ*slr0320* mutant grown under NL and HL. (**a**) Volcano plots showing differentially expressed proteins in WT and the Δ*slr0320* mutant grown under NL and HL; (**b**) bubble plot showing enrichment of significantly upregulated proteins in the Δ*slr0320* mutant grown under NL by fisher’s exact test. The bubble size indicates the number in each term; bubble color indicates the *p* value as shown in the scale bar above the plot.

The cutoffs mentioned above were applied to filter proteins of WT and the Δ*slr0320* mutant grown under NL and HL as well. Compared to WT under NL, 26 and 18 proteins were significantly down- and upregulated in the Δ*slr0320* mutant under NL, respectively (Figure 6a and Table S3). Enrichment of those significantly upregulated proteins by Fisher’s exact test revealed that Slr0320 had effects on PSII (Figure 6b and Table S6). One of the low molecular weight subunits of PSII [44], PsbL, was significantly upregulated (Figure 6a). Compared to WT under HL, there are 54 downregulated proteins and 28 upregulated proteins in the Δ*slr0320* mutant under HL (Figure 6a and Table S4). Intriguingly, the significantly upregulated effect on PSII in the Δ*slr0320* mutant grown under NL disappeared when grown under HL. For all identified PSII subunits, none was significantly differentially expressed (Figure 6a and Table S4). Likewisely, these proteomic data were further confirmed by our immune detections (Figure 5b).

### 3.5. Expression of Low Molecular Weight Subunits of PSII in WT, Δslr0320 and PpetJ::slr0320 Strains

**Figure 7:**
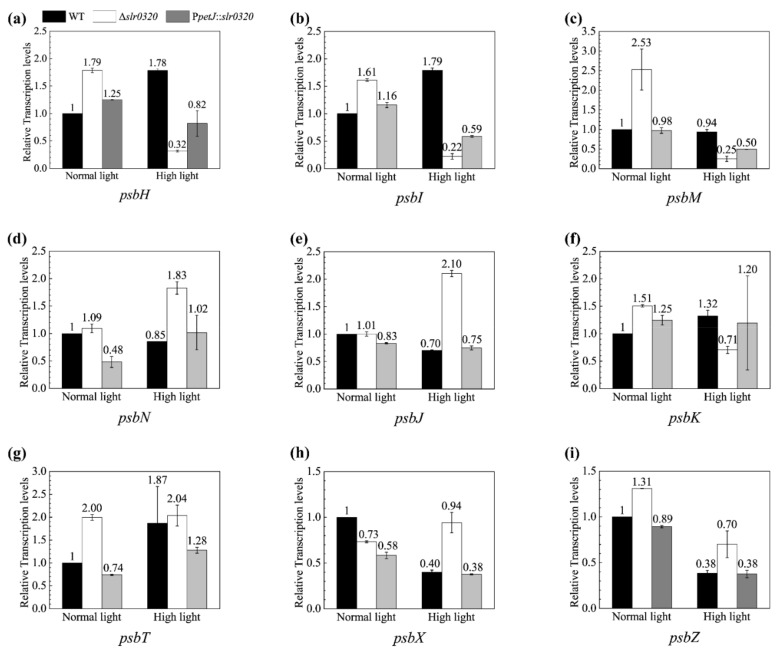
Transcript accumulation of genes encoding 9 low molecular weight (LMW) subunits of PSII in WT and the Δ*slr0320* mutant. (**a**) transcription accumulation of *psbH* in WT and the Δ*slr0320* mutant grown under NL and HL; (**b**) transcription accumulation of *psbI* in WT and the Δ*slr0320* mutant grown under NL and HL; (**c**) transcription accumulation of *psbM* in WT and the Δ*slr0320* mutant grown under NL and HL; (**d**) transcription accumulation of *psbN* in WT and the Δ*slr0320* mutant grown under NL and HL; (**e**) transcription accumulation of *psbJ* in WT and the Δ*slr0320* mutant grown under NL and HL; (**f**) transcription accumulation of *psbK* in WT and the Δ*slr0320* mutant grown under NL and HL; (**g**) transcription accumulation of *psbT* in WT and the Δ*slr0320* mutant grown under NL and HL; (**h**) transcription accumulation of *psbX* in WT and the Δ*slr0320* mutant grown under NL and HL; (**i**) transcription accumulation of *psbZ* in WT and the Δ*slr0320* mutant grown under NL and HL. The abundances of the transcripts were calculated relative to the reference gene *rnpB*. The transcripts of WT grown under NL were set as 1. The transcripts of WT grown under HL and mutant grown both under HL and NL were calculated relative to the WT grown under NL. Numbers above bars indicate the mean of relative transcription levels. The results are shown as the means from three independent experiments ± SD.

Besides PsbLthat the only significantly upregulated PSII subunit in the Δ*slr0320* mutant was a low molecular weight (LMW) subunit, it is worth analyzing the expression of other LMW subunits. Of 13 LMW PSII subunits, our proteomic study identified four of them: PsbE, PsbF (Smr0006), PsbY (Sml0007) and PsbL. Except PsbL, the other three did not show significant changes in expression between WT and the Δ*slr0320* mutant (Figure 6a and Tables S3 and S4). Many attempts to separate and immuno-detect these LMW subunits by SDS-PAGE and Western blot failed, probably due to their nature of low protein mass. Therefore, transcriptional levels of the remaining nine LMW proteins detected by quantitative real-time PCR (qRT-PCR) were analyzed (Figure 7).

As shown in Figure 7, the expression patterns of *psbH* (*ssl2598*) (Figure 7a) and *psbI* (*sml0001*) (Figure 7b) in the Δ*slr0320* mutant were highly sensitive to HL. The transcripts of these two genes were slightly upregulated in the Δ*slr0320* mutant under NL, but markedly downregulated by more than five times compared to WT under HL. Compared to WT and the P*petJ*::*slr0320* strain grown under HL, the *psbM* (*sml0003*) (Figure 7c) and *psbK* (*sml0005*) (Figure 7f) genes in the Δ*slr0320* mutant grown under HL were only slightly downregulated—i.e., by less than four times. Unlike the downregulations of *psbH*, *psbI*, *psbK* and *psbM* in the Δ*slr0320* mutant grown under HL, the *psbN* (*smr0009*) (Figure 7d), *psbJ* (*smr0008*) (Figure 7e), *psbT* (*smr0001*) (Figure 7g), *psbX* (*sml0002*) (Figure 7h) and *psbZ* (*sll1281*) (Figure 7i) were upregulated in the Δ*slr0320* mutant compared to WT and P*petJ*::*slr0320* strains grown under HL.

## 4. Discussion

### 4.1. Slr0320 Is Crucial for Cyanobacteria during HL Acclimation

Slr0320 protein is conserved among sequenced cyanobacteria. Although Slr0320 was nonresponsive to HL in *Synechocystis* WT (Figure 5a), inactivation of *slr0320* led to a significant defect in the photosynthetic growth under HL (Figure 1b,c). The growth difference between WT and the Δ*slr0320* mutant under NL was not significant (Student’s *t* test, *p* < 0.05), suggesting a more important role of Slr0320 under the HL condition. The fact that reconstitution of the *slr0320* gene at a neutral site of the genome in the Δ*slr0320* mutant restored the growth of the insertional mutant under HL further confirmed the growth phenotype by inactivation of *slr0320* (Figure 1b,c). That the net photosynthesis rate measured in the presence of bicarbonate was significantly decreased (Student’s *t* test, *p* < 0.05) in the Δ*slr0320* mutant grown under HL (Table 2) may explain the retarded growth. The standard condition commonly used in most laboratories for growing *Synechocystis* is under 50 µmol photons m^−2^ s^−1^, whereas here we used NL conditions. Elevation of light intensity moderately to a sub-saturating condition increased the growth rate of WT due to more light energy. This HL condition, however, decreased the growth rate of the Δ*slr0320* mutant (Figure 1b,c). These data suggest that moderately high light could not be used as additional light substrate for photosynthesis but was harmful to the cells when Slr0320 was missing. This phenomenon promoted us to figure out how Slr0320 affects photosynthesis during HL acclimation.

### 4.2. Slr0320 Is Involved in Optimal Function of PSII

Despite poor growth of the Δ*slr0320* mutant under HL (Figure 1b,c), the accumulation of major photosynthetic proteins of thylakoid membranes had little change among three strains (Figure 2a). Similar to the protein amounts, impacts of the interruption of *slr0320* on the activities of PSI and the Calvin–Benson cycle may not dramatically change. The measurements of P700 oxidation-reduction detected by absorbance changes in WT and the Δ*slr0320* mutant showed that, illuminated by saturated far-red light (725 nm, 1400 μmol photons m^−2^ s^−1^), which solely activates PSI and in the presence of methyl viologen which accepts electrons directly from acceptor side of PSI [45], the oxidation and subsequent reduction traces of the Δ*slr0320* mutant were very similar to those of WT grown under NL and HL (Figure S1), indicating that the electron transfer within PSI was normal in the Δ*slr0320* mutant. Previous reports showed that the activity of the Calvin–Benson cycle was positively correlated to protein amounts of RuBisCo, SBPase, aldolase and transketolase in plants and *Synechocystis* [46,47]. Our proteomic data show that these proteins were not significantly differentially regulated in the mutant (Tables S3 and S4), suggesting the activity of the Calvin–Benson cycle in the Δ*slr0320* mutant could be similar to WT. In addition to the similar PSII accumulation (Figure 2a), the assembly of PSII complexes in the Δ*slr0320* mutant was not affected either (Figure 2b). The HL acclimation responses of *Synechocystis* cause efficient PSII photorepair. Previous reports revealed that this process involves FtsH proteases [48], EF-Tu, EF-G [19], Slr1051 [21], CrtO [18], Psb28 and Psb27 [43] and the efficiency of PSII photorepair under increasing light intensity is strongly associated with the amounts of these proteins. Our proteomics of WT grown under NL and HL revealed that these proteins were significantly differentially regulated under HL, further supporting that the efficient PSII photorepair is closely associated with their amounts (Figure 5a). Our proteomic data of the Δ*slr0320* mutant grown under NL and HL show that the amounts of these PSII photorepair related proteins were not significantly changed in the Δ*slr0320* mutant (Figure 6a and Tables S3 and S4), indicating the photorepair and assembly of PSII complex under HL were unlikely the major reasons for the HL phenotype of the Δ*slr0320* mutant. Chlorophyll fluorescence traces showed that the Δ*slr0320* mutant had much higher stable fluorescence Fs than that of the WT but almost normal Fo (Figure 3a,b), suggesting higher portion of closed PSII centers under light in the mutant. These data suggest a defect in electron transfer processes at the acceptor side of PSII in the Δ*slr0320* mutant. To confirm this, flash-induced chlorophyll fluorescence measurements were performed (Figure 3c). Illuminations of *Synechocystis* by a single turn over flash could result in an increase in the chlorophyll fluorescence yield simultaneously [36]. The subsequent decay of fluorescence yield in the dark reflects three electron transfer pathways of Q_A_^−^ reoxidation. First, the fast electron transfer from Q_A_^−^ to Q_B_ is accomplished by a plastoquinone molecule already bound to the Q_B_ pocket at the moment of the flash. Second, the middle phase resembles the Q_A_^−^ oxidized by free PQ from the PQ pool in the thylakoid membrane diffusing to the Q_B_ pocket. Third, the slow phase resembles the Q_A_^−^ reoxidized by the S_2_ state of water-oxidizing complex. The fluorescence decay was much slower in the Δ*slr0320* mutant, especially when the cells were grown under HL (Figure 3c and Table 3), indicating that, in the absence of Slr0320 protein, the Q_A_^−^ reoxidation process is slowed down. The major reasons for the slow Q_A_^−^ reoxidation were smaller amplitude of fast phase and bigger time constant of fast phase and middle phase. These data suggest that the acceptor side of PSII was modified in the Δ*slr0320* mutant. Further oxygen evolution measurements in the presence of DCBQ (Table 2) suggest that, when enough quinone acceptor was provided, the PSII in the Δ*slr0320* mutant were as functional as those in the WT. Therefore, we assumed that there was probably a defect in the quinone-binding of PSII due to the lack of Slr0320. Despite this, we identified effects of the interruption of *slr0320* on the PSII acceptor side and the activities of PSI and the Calvin–Benson cycle were unchanged, so it would be unwise to exclude the possibility that Slr0320 still has an impact on other photosynthetic components downstream of PSII, such as cytochrome *b*_6_*f*. When the downstream of photosynthetic electron transport is inhibited, the PQ pool could stay reduced and thus the Fs increases [49], as shown in Figure 3a,b. The proteomics data and Western blots (Figure 5b and Figure 6a; Tables S3 and S4) showed that the expression of subunits of the cytochrome *b*_6_*f* complex was not altered in the Δ*slr0320* mutant under both NL and HL, suggesting that it may not the major reason for the obvious phenotype.

It has been reported that some low molecular weight (LMW) subunits of PSII may affect assembly and quinone-binding [6,8]. Indeed, one of those LMW subunits, PsbL, was found to be significantly upregulated in the Δ*slr0320* mutant from our proteomic data (Figure 6a and Table S3). Previous reports have demonstrated that PsbL is a key factor for stabilization of PSII complexes and, by potential indirect interaction with D1 protein, influences PSII structure and the Q_A_ activity [43,49,50]. A detailed investigation on tobacco PsbL revealed that additional PsbL protein could optimize the binding of plastoquinone with the PSII complex and subsequent electron transfer in vitro [50]. Given that PsbL is highly conserved between *Synechocystis* and plants [8], the role of PsbL could be similar in *Synechocystis*. The enhanced expression of PsbL in the Δ*slr0320* mutant under NL may function in optimizing the binding of PQ to PSII and further partially restore the PSII electron transfer. This could be the reason that the impaired electron transfer from Q_A_ to Q_B_ in the Δ*slr0320* mutant was less evident under NL than HL (Figure 3c and Table 3). Among 13 LMW subunits of *Synechocystis*, PsbE, PsbF, PsbL and PsbY were also detected but no significant difference was shown between WT and the Δ*slr0320* mutant by proteomic analysis under HL (Figure 6a and Tables S3 and S4). Due to failure in the proteomic identification and difficulties in protein-level detection, we applied qRT-PCR to examine the expression of remaining nine LMW subunits. The data showed that *psbH* and *psbI* were downregulated by more than five times and could possibly have effects on their protein levels in the Δ*slr0320* mutant under HL (Figure 7). Though *psbM* and *psbK* were downregulated as well, we thought their transcriptional downregulations might be minor. Due to the retarded photoautotrophic growth rates, highly sensitive to high light and slower electron transfer within PSII in *psbH* and *psbI* inactivation mutants, these two genes were considered to regulate electron transfer from Q_A_ to Q_B_ [51,52,53,54,55,56]. Furthermore, multiple reports of the crystal structure of PSII suggested that these two genes products were highly related to the acceptor-side structure within PSII [1,2,3]. Therefore, we thought that the much more evident impaired electron transfer from Q_A_ to Q_B_ caused by the interruption of *slr0320* under HL was possibly due to decreases in *psbH* and *psbI*.

The B12-binding and the radical SAM domains of Slr0320 resemble the class B radical SAM methyltransferases. Previous reports have demonstrated that the radical SAM methyltransferases use SAM and other cofactors, such as B12 (cobalamin), to catalyze methylations. Members of the radical SAM methyltransferases were more than 113,000 and identified to methylate a wide variety of natural products, playing very important roles [57,58,59,60]. RlmE from various microorganisms, such as *Escherichia coli* [61,62] and *Saccharomyces cerevisiae* [63,64], and RlmN from *Escherichia coli* [65] revealed that the radical SAM methyltransferases were responsible for tRNA methylation and the subsequent ribosome assembly. The Fom3 from *Streptomyces wedmorensis* was identified to catalyze methylations in the biosynthesis of antibiotic fosfomycin [66]. In addition to the substrates mentioned above, many reports have shown that some members of this superfamily from various microorganisms, such as MenA and MenG from *Escherichia coli* and *Streptomyces coelicolor*, MenK from *Adlercreutzia equolifaciens*, *Collinsella tanakaei* and *Ferrimonas marina*, were involved in the methylation of menaquinone and composition of the PQ pool, and this adenosylmethionine-dependent methylation had important roles on the biosynthesis and function of quinone [67,68,69,70]. Considering that the impaired electron transfer from Q_A_ to Q_B_ (Figure 3c) might be due to the poor binding of PQ to PSII in the Δ*slr0320* mutant, it cannot be excluded that the Slr0320 is a member of the radical SAM methyltransferases, catalyzing the methylation of quinone, and the interruption of *slr0320* may result in defects of methylation of quinone, affecting its binding to PSII and impairing electron transfer between Q_A_ and Q_B_. Yet, more proofs still need to be discovered.

Combining all the data above, a conclusion is made. Other than PSII photorepair and assembly, Slr0320 is involved in the electron transfer within PSII during HL acclimation of *Synechocystis.* The interruption of *slr0320* leads to an impaired electron transfer from Q_A_ to Q_B_ within PSII. This impaired electron transfer could be partially restored by upregulation of PsbL under NL, possibly enhancing the binding of plastoquinone and consequently maintaining the mutant to grow similar to WT. However, the impaired electron transfer was further inhibited under HL, probably due to downregulations of *psbH* and *psbI* and defects in the structure of the acceptor side. Based on the conserved domains, there is a hint that Slr0320 might be involved in methylation of plastoquinone, further affecting the binding to PSII, and electron transfer from Q_A_ to Q_B_ cannot be excluded; more proofs are needed.

## Figures and Tables

**Table 1:** Primers used in this assay.

	Forward Sequence (5′–3′)	Reverse Sequence (5′–3′)
P4	CGACGGGCAATTTGCACTTCAG	CGTATTAGCTTACGACGCTACACCC
*psbH* (*ssl2598*)	GATATCCTCAGACCCCTCAAC	CAGAAAAAGAGCCATAAATACCC
*psbI* (*sml0001*)	CCCTTAAAATCGCCGTTT	CAAAGTCTTTGCGGCCAG
*psbM* (*sml0003*)	ACAATCTCGGCTTTATAGCAAG	CCCGGTTTGAATAAACAGGATC
*psbK* (*sml0005*)	GGAAACAATTTATTTGCTCGC	AAAAGAAGAGGGGAATGACCG
*psbX* (*sml0002*)	TGACCCCTTCTTTAGCAAACT	CTGATGAAAATTAACCCGACG
*psbZ* (*sll1281*)	TCAGCGACTGTCGAGGAT	GCCAACGCAATCTGAAAA
*psbJ* (*smr0008*)	ATGTTCGCAGAAGGCAGAATC	ACCAGCATAGGCTCCGTAGAA
*psbN* (*smr0009*)	TCCGCAACAGTTCTTAGCAT	GTCATCGAAGGGATCACCCA
*psbT* (*smr0001*)	ATGGAAAGTGTTGCTTACATTCTG	CTATTTTTCGATGCGGGGG
*rnpB* (*slr0249*)	TTTAGAAAACAGCAACCAGT	GGCAGGAAAAAGACCAACCT

**Table 2:** Steady-state oxygen evolution of WT, Δ*slr0320* and P*petJ*::*slr0320* strains grown under NL and HL in the presence of NaHCO_3_ and DCBQ.

Strains and Growth Conditions	Oxygen Evolution (μmol O_2_ mg^−1^ Chlorophyll h^−1^)	
	H_2_O to CO_2_ (NaHCO_3_)	H_2_O to Quinone (DCBQ)
Normal light		
WT	311 ± 8	467 ± 16
Δ*slr0320*	358 ± 12	512 ± 13
P*petJ*::*slr0320*	330 ± 13	478 ± 17
High light		
WT	435 ± 14^a^	538 ± 12
Δ*slr0320*	335 ± 16	607 ± 15
P*petJ*::*slr0320*	444 ± 10^b^	508 ± 9

Student’s *t* test was applied. The superscript “a” marks significant differences for WT grown under HL vs. grown under NL in the presence of NaHCO_3_; the superscript “b” marks significant differences for the complementary P*petJ*::*slr0320* grown under HL vs. grown under NL in the presence of NaHCO_3_, both with a significance level of *p* < 0.05 The results are shown as the means from three independent experiments ± SD.

**Table 3:** Kinetics of the flash-induced chlorophyll fluorescence of WT, Δ*slr0320* and P*petJ*::*slr0320* strains under NL and HL.

Strains and Growth Conditions	Fast Phase		Middle Phase		Slow Phase	
	T1 (ms)	A1 (%)	T2 (ms)	A2 (%)	T3 (s)	A3 (%)
Normal light						
WT	619.65 ± 21.70	60 ± 1.0	5.8 ± 0.62	23 ± 0.9	6.1 ± 1.1	18 ± 1.1
Δ*slr0320*	880.02 ± 22.01	51 ± 1.5	13.1 ± 0.77	27 ± 0.4	4.0 ± 0.5	23 ± 1.3
P*petJ*::*slr0320*	624.24 ± 24.62	60 ± 1.9	7.5 ± 0.96	21 ± 1.0	5.0 ± 0.6	19 ± 1.3
High light						
WT	709.52 ± 62.49	53 ± 2.9	6.0 ± 1.33	25.83 ± 2.0	4.4 ± 0.2	21 ± 2.4
Δ*slr0320*	1366.25 ± 131.58	25 ± 3.6	15.9 ± 2.09	34.82 ± 0.6	1.9 ± 0.3	40 ± 3.2
P*petJ*::*slr0320*	779.81 ± 60.90	54 ± 2.5	8.6 ± 1.47	22.36 ± 2.3	4.2 ± 0.4	23 ± 1.8

The results are shown as the means from three independent experiments ± SD.

## Data Availability

The reported ion intensities of quantitative proteomics presented in this study are available in Table S1.

## Supplementary Materials

The following are available online at https://www.mdpi.com/article/10.3390/life11040279/s1, Figure S1: P700 oxidation-reduction in WT and the Δ*slr0320* mutant grown under NL and HL; Figure S2: The mutant segregation by genomic PCR; Table S1: Reported ion intensities of quantitative proteomics; Table S2: Names of significantly differential expressed proteins involved in HL acclimation of *Synechocystis*; Table S3: Names of significantly differential expressed proteins of WT and Δ*slr0320* grown under NL; Table S4: Names of significantly differential expressed proteins of WT and Δ*slr0320* grown under HL; Table S5: All Fisher’s exact test results of significantly differential expressed proteins involved in HL acclimation of *Synechocystis*; Table S6: All Fisher’s exact test results of significantly differential expressed proteins of WT and Δ*slr0320* grown under NL.
