# Supplementary material for: Slr0320 Is Crucial for Optimal Function of Photosystem II during High Light Acclimation in Synechocystis sp. PCC 6803
Source: Life (Basel). 2021 Mar 26;11(4):279. doi: 10.3390/life11040279 (PMC8065906; doi:10.3390/life11040279)
Supplement: Supplementary file 1 [file life-11-00279-s001.zip › Figure S1, S2.docx]

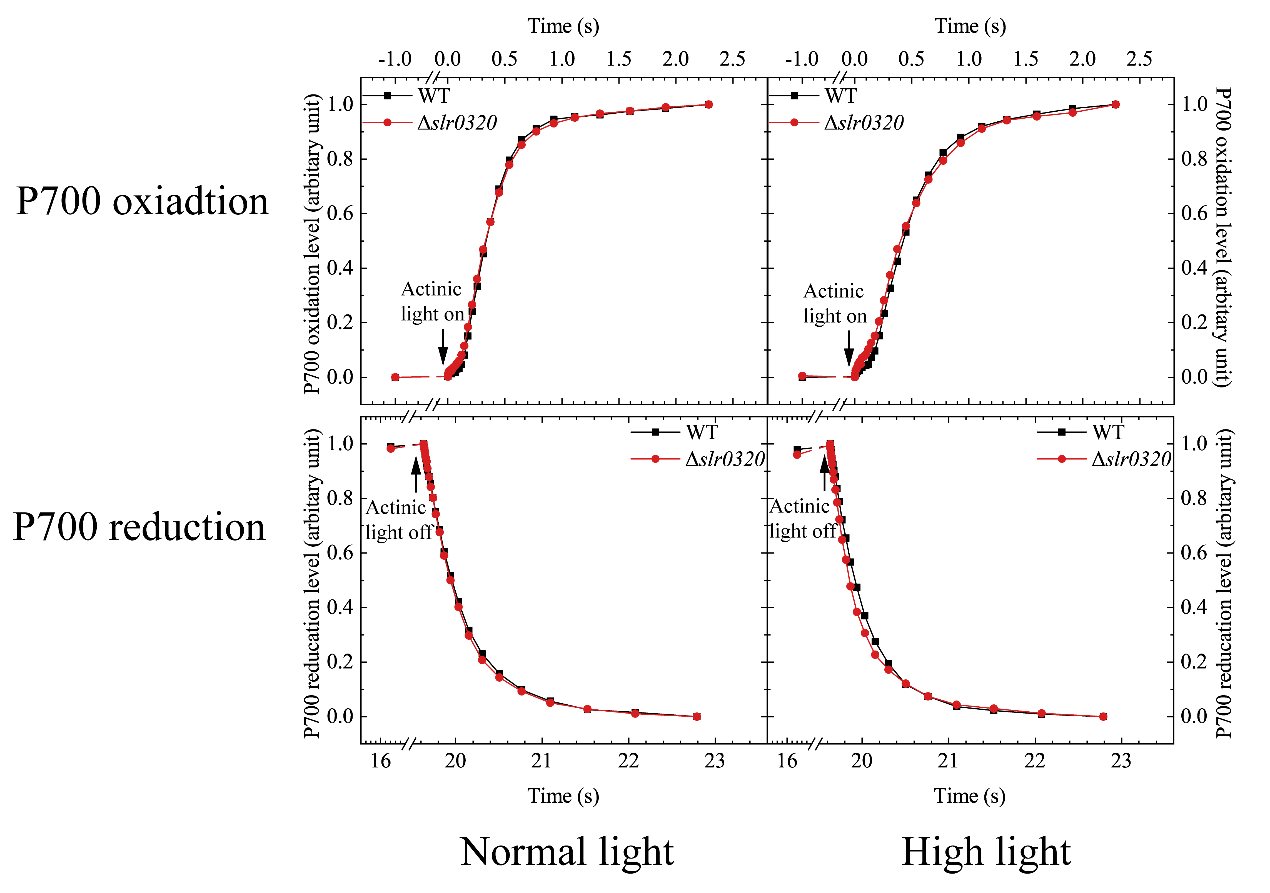


**Figure S1.** P700 oxidation-reduction in WT and the Δ*slr0320* mutant grown under NL and HL. Dark adapted cells at final chlorophyll concentration 10 μg/ml were illuminated by far-red light (725 nm, 1400 μmol photons m^-2^ s^-1^) for 20 s and followed by darkness. The absorbance changes at 810 nm were monitored by dark-pulse mode using Jts-10 (Biologics). To eliminate cyclic electron flow, which affects P700+ reduction, 200 μM methyl viologen was supplemented in the cell suspension during the measurements.


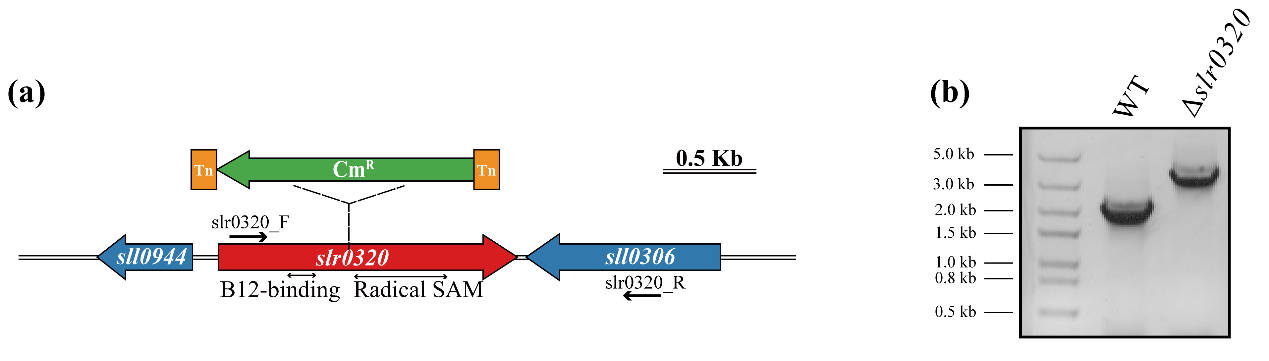


**Figure S2.** The mutant segregation by genomic PCR. (a) The localizations of primers for the genomic PCR by slr0320_F (5’- TTCGTCTTCCCTGTAACCCG -3’) and slr0320_R (5’- GCCTACTGGTGGATTCGCC -3’); (b) Segregation of *slr0320* in WT and the Δ*slr0320* mutant. After 40 cycles of PCR, none of the wild-type *slr0320* was detected in the Δ*slr0320* mutant.
